# Significant sequelae after bacterial meningitis in Niger: a cohort study

**DOI:** 10.1186/1471-2334-13-228

**Published:** 2013-05-21

**Authors:** Jean-François Jusot, Zilahatou Tohon, Abdoul Aziz Yazi, Jean-Marc Collard

**Affiliations:** 1 Epidemiology/Health-Environment-Climate Unit, Centre de Recherche Médicale et Sanitaire, PO Box 10887, Niamey, Niger; 2 Epidemiolgy DrPH Candidate, College of Public Health, University of Kentucky, 111 Washington Avenue, Lexington, KY 40536-0003, USA; 3 Epidemiology Unit, Epicentre, PO Box 13330, Niamey, Niger; 4 Biology Unit, Centre de Recherche Médicale et Sanitaire, PO Box 10887, Niamey, Niger

**Keywords:** Meningococcal, Complications, Disability, Less-developed countries

## Abstract

**Background:**

Beside high mortality, acute bacterial meningitis may lead to a high frequency of neuropsychological sequelae. The Sahelian countries belonging to the meningitis belt experience approximately 50% of the meningitis cases occurring in the world. Studies in Africa have shown that *N*. *meningitidis* could cause hearing loss in up to 30% of the cases, exceeding sometimes measles. The situation is similar in Niger which experiences yearly meningitis epidemics and where rehabilitation wards are rare and hearing aids remain unaffordable. The aim of this study was to estimate the frequency of neuropsychological sequelae after acute bacterial meningitis in four of the eight regions of Niger.

**Methods:**

Subjects exposed to acute bacterial meningitis were enrolled into a cohort with non exposed subjects matched on age and gender. Consenting subjects were interviewed during inclusion and at a control visit two months later. If clinical symptoms or psychological troubles persisted at both visits among the exposed subjects with a frequency significantly greater than that observed among the non exposed subjects, a sequelae was retained. The comparison of the frequency of sequelae between non exposed and exposed subjects to bacterial meningitis was also calculated using the Fisher exact test.

**Results:**

Three persisting functional symptoms were registered: headaches, asthenia, and vertigo among 31.3, 36.9, and 22.4% respectively of the exposed subjects. A significant motor impairment was retrieved among 12.3% of the exposed versus 1.6% of the non exposed subjects. Hearing loss significantly disabled 31.3% of the exposed subjects and 10.4% exhibited a serious deafness.

**Conclusions:**

This study carried out in Niger confirms two serious neurological sequelae occurring at high frequencies after bacterial meningitis: severe and profound hearing loss and motor impairment. Cochlear implantation and hearing aids are too expensive for populations living in developing countries. Neurological sequelae occurring after meningitis should sensitize African public health authorities on the development of rehabilitation centers. All these challenges can be met through existing strategies and guidelines.

## Background

Beside high mortality, acute bacterial meningitis may lead to a high frequency of neuropsychological sequelae in 3 to 47% of cases, causing 160,000 yearly disabilities worldwide [1-5]. Among the three main microorganisms causing acute bacterial meningitis, *Streptococcus pneumoniae* (Sp) is the most lethal and most disabling followed by *Haemophilus influenzae* (Hi) and *Neisseria meningitidis* (Nm) [6].

The Sahelian countries belonging to the meningitis belt experience approximately 50% of meningitis cases for a population at risk with an estimated 350 million inhabitants. Indeed, epidemics occur every year with major epidemics every 5 – 12 years with high attack rates. Attack rates are high ranging from 100 to 800 per 100,000. Since the first cerebrospinal meningitis outbreak occurred in Nigeria in 1905, other epidemics have threatened the meningitis belt [7]. In 1996, large outbreaks spread across the meningitis belt leading to 250,000 suspected cases and 25,000 deaths [8]. They were mainly due to *N*. *meningitidis* serogroup A. In 2002 a large outbreak due to *N*. *meningitidis* W occurred in Burkina Faso [9]. Again in 2009, 80,000 cases and more than 4,000 deaths occurred in this part of Africa [10]. Niger experienced large outbreaks in 2000, 2003 and 2009, in which *N*. *meningitidis* serogroup A was the main causative agent. Although a polysaccharide vaccine (A, C, W, Y serogroups of *N*. *meningitidis*) was widely used in mass vaccination campaigns to control transmission of the disease, a new conjugate vaccine against serogroup A was introduced in 2010. Other emerging serogroups of *N*. *meningitidis*, such as W135 and X, have been incriminated in recent outbreaks [11,12].

In the perspective of physical rehabilitation, the most serious sequelae after acute bacterial meningitis are neurological including motor impairment, epilepsy, cecity or vision loss, speech disorder and hearing loss. In Africa, studies of bacterial meningitis sequelae have been mainly conducted more within hospital wards than in peripheral and remote health care centres usually located far from reference hospitals. The majority of the studies on post-meningitis sequelae therefore consider in-hospital outcomes whereas post-discharge outcomes are less frequently reported [4].

Hearing loss could result in irreversible neurological sequelae, frequently severe or profound, probably due to a serious or suppurative labyrinthitis occurring very early during the acute phase of meningitis and evolving towards a labyrinthitis ossificans [13,14]. The frequency of hearing loss exhibits large variations, between 2 and 48% depending on the studies [4]. Pneumococci have been documented as the major cause of severe/profound hearing loss leading to the majority of cochlear implantations [15]. Nevertheless, studies in Africa have shown that *N*. *meningitidis* could be the cause in 30% of hearing loss, sometimes exceeding measles [16-18]. The situation is similar in Niger which experiences yearly meningitis epidemics and yet where rehabilitation wards are rare and hearing aids remain unaffordable and too expensive for the disabled. In addition, frequency of sequelae after bacterial meningitis has not yet been rigorously determined in the country. Consequently, it is difficult to estimate the need for rehabilitation and sensitization for health authorities without rigorous data for decision making.

The aim of this study was therefore to estimate the frequency of the sequelae after acute bacterial meningitis in four of the eight regions of Niger.

## Methods

### Study population

The study population concerned inhabitants of four of the eight regions of Niger: the capital city Niamey, Dosso, Tillabery, and Maradi. These four regions comprised 9 million among the 15 million inhabitants of Niger in 2011.

From 27/12/2010 - 13/05/2011, 252 cases could be potentially enrolled in the regions targeted by this study, representing 87.3% due to *N*. *meningitidis* of W serogroup and 11.5% of *S*. *pneumoniae*.

### Study design

The study design consisted of an exposed/non exposed cohort. Eligible subjects were retrieved after study interviewers inquired from the district health authorities if suspected meningitis cases had been notified. Identified subjects were considered exposed if one of the three main causal agents, *N*. *meningitidis*, *S*. *pneumoniae* or *H*. *influenzae*, was identified in their cerebral spinal fluid (CSF) by PCR or latex test performed as part of the routine microbiological surveillance coordinated by the CERMES. Non exposed subjects did not have any meningitis or meningeal syndrome according to the WHO suspected case classification [19]. They were selected from the family of their corresponding exposed subject and matched on gender and age. Subjects known to have suffered from a similar pathology as the post-meningitis sequelae were excluded. All the subjects were enrolled if they were 5 years old and above to facilitate the clinical and audiometric examination.

### Studied factors

After enrolment, all the exposed and non exposed subjects were interviewed by two physicians, one located at Niamey and the other at Maradi. Both physicians were readily available to perform an immediate visit when a case was suspected or diagnosed in a health care facility. They were therefore aware of the infectious status of the subjects visited at the health care facility during their acute clinical phase for the exposed subjects. Non exposed subjects were visited at home. The two physicians were general practitioners trained on the aims of the study and the tools to be used (protocol, operational guide, questionnaires, audiogram, consent form). They performed a complete and in-depth clinical examination, especially focused on seeking neurological, sensitive, sensorial, and motor impairments. Clinical and psychological examinations as well as audiograms (Amplitude® T-Series audiometer) were performed both at the inclusion visit and during the control visit performed 2 months later. This duration of follow-up was validated by the scientific committee of the study due to the difficulties to retrieve subjects living in remote and difficult-to-access areas.

For the audiology study, the audiograms were interpreted by two physicians specialised in otorhinolaryngology who were blinded to the exposed or non exposed status of the subjects. The hearing losses were classified according to the classification of the Bureau International d’Audiophonologie. Deafness was mild, moderate, severe or profound when corresponding losses were from 21 to 40 dB, 41to 70 dB, 71 to 90 dB, and >90 dB respectively. Severe and profound hearing losses were regrouped under serious hearing loss. Air and bone conduction was also assessed to identify the type of hearing loss.

Psychological behaviour was assessed by two psychologists during the visits with the physicians. The two psychologists belong to the technicians’ category of the civil service in Niger. The following items extracted from the Conners’questionnaire were explored: blue mood, decreased energy, decreased of pep, poor motivation, serious asthenia, irritability, scared without any reason, sleeping difficulties, difficulty concentrating, nervousness, crying without any reason, and difficulty in daily living activities.

If the clinical symptoms or psychological disorders persisted at the control visit among the exposed subjects with frequencies significantly greater than those observed among the non-exposed subjects, a sequelae was retained. The audiogram result for the most altered ear at the control visit was retained to qualify the level and type of hearing loss [20].

All the findings were recorded in a questionnaire. All the questions were asked in the local language when necessary.

### Statistical analysis

The statistical analysis estimated the frequency of the symptoms at inclusion between exposed and non-exposed subjects. The comparison was made using the Fisher exact test. In order to show a trend in the severity of hearing loss associated to bacterial meningitis, a trend test of Cochran-Armitrage was used. These two tests were performed using the software Winpepi [21].

A score for behavioural troubles was built and validated by estimating the reliability of 12 psychological items using the packages ltm and CMC of R software to estimate Cronbach alpha coefficient [22]. The presence of an item accounted for 1 point versus 0 in its absence. The score was calculated by summing the points with the retained items.

The comparison of the frequency of the sequelae between exposed and non exposed subjects was also calculated using the Fisher exact test. All the frequencies were estimated with a 95% confidence interval.

### Ethical considerations

Study information was provided to the subjects or to their family. Written informed consent was obtained before inclusion. The study protocol was approved by the National Ethics Committee of Niger (authorisation N°014//2009/CCNE).

## Results

### Characteristics of the subjects included

**Figure 1:**
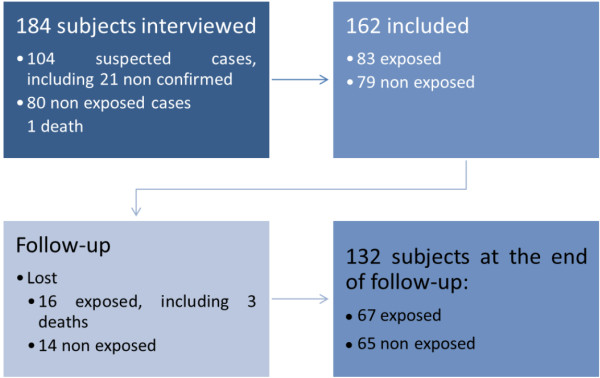
Flow chart explaining the distribution of the subjects during follow-up and according to their status.

Among the 184 subjects who met enrolment criteria, 104 suspected as clinical acute bacterial meningitis were visited and 83 were confirmed as exposed following biological confirmation of acute bacterial meningitis. Seventy nine non exposed subjects were included after one died. During the follow-up, 3 exposed subjects died (case fatality rate = 4.3%), and 13 exposed (15.7%) and 14 non exposed (17.7%) subjects were lost to follow-up two months after the inclusion visit. Finally, 67 exposed and 65 non exposed subjects were retained for the analysis to estimate the frequency of the sequelae. (Figure 1) The subjects were visited 87 days (range = 50 – 141 days) on average after their inclusion visit.

### Symptoms associated with meningitis

At inclusion, the 162 subjects recruited in the study consisted of 83 meningitis cases and 79 non exposed subjects. There was no gender predominance in the two groups. The mean age was 13.5 years (SD = 8.9 y, range = 5 – 60 years) without any difference between meningitis cases and non exposed subjects. The most frequent age groups were 5 – 14 years and 15 – 24 years with respectively 69.8% and 22.2% of the subjects. The predominant causal agent was *N*. *meningitidis* W in 86.7% of the meningitis cases; 6% were due to *S*. *pneumoniae*, 6% to *N*. *meningitidis* serogroup A and 1.2% to serogroup C (Table 1).

**Table 1:** Basic parameters on the included subjects

**Parameter**	**Exposed**	**Non exposed**
	**n = 83**	**n = 79**
Age: mean (SD)	13.7 (10.1)	13.2 (7.55)
Sex:		
- Male: n (%)	44 (53.0)	38 (48.1)
Etiological agent by PCR or Latex:		
- N. *meningitidis*: n (%)	78 (94)	0
Serogroup W135	72 (86.7)	0
Location:		
- Niamey: n (%)	53 (63.9)	52 (65.8)

The main symptoms associated with meningitis are depicted in Table 2 and comprised functional symptoms like headache, asthenia, and insomnia in at least 50% of the exposed subjects. Motor impairment for tonus and walking was significantly found among 78.8% of the exposed subjects in contrast to 5.1% of non exposed subjects. Odour perception for soumbala, a well known traditional spice, was altered for 45.4% of bacterial meningitis cases. Facial muscles, bladder and anal sphincter were also impaired in respectively 17.5, 31.3 and 43.8% of the bacterial meningitis cases. No cecity was encountered, but the mean of visual acuity among meningitis cases was lower than for the non exposed subjects with respectively 8.8 and 9.3 (t test = −2.33, df = 128.2, p = 0.02). Only bone conduction or both bone and air conductions and serious hearing losses were predominant during acute bacterial meningitis, with OR = 4.84 and 13.1 respectively.

**Table 2:** Symptoms associated with meningitis at the inclusion

**Symptoms**	**Exposed**		**Non exposed**		**OR**	**95% CI**		**p**
	**n = 83**	**%**	**n = 79**	**%**	**Crude**	**Lower**	**Upper**	
Headaches	79	59.5	79	29.1	3.6	1.8	7.1	0.0002
Insomnia	80	58.8	79	22.8	4.8	2.3	10.2	<0.0001
Convulsions before admission	80	83.8	78	5.1	90.9	27.8	400	<0.0001
Asthenia	79	78.5	79	38.0	5.9	2.8	12.8	<0.0001
Sensation to pain and to touch perturbed	80	13.8	78	0	NC	2.7	NC	0.0007
At least one troubled reflex	80	62.5	78	15.4	9.0	4.1	21.3	<0.0001
Walking or tonus impaired	80	78.8	79	5.1	66.7	20.8	286	<0.0001
Odor of soumbala non recognised	77	45.4	77	22.1	2.9	1.4	6.3	0.004
Masseter motility impaired	80	21.3	78	0	NC	4.8	NC	<0.0001
Facial motility impaired	80	17.5	77	3.9	5.2	1.4	29.4	0.009
Trouble in the voice	80	32.5	76	1.3	35.7	5.5	1493	<0.0001
Vertigo	79	55.7	79	15.2	6.9	3.1	16.4	<0.0001
Hearing loss type	76		79					0.0002
- No	27	35.5	53	67.1	1			
- Air conduction altered	12	15.8	11	13.9	2.14	0.8	6.1	0.14
- Bone conduction altered or mixed	37	48.7	15	19.0	4.84	2.1	11.2	<0.0001
Hearing loss level	78		79					<0.0001
- No	27	34.6	53	67.1	1			
- Mild	20	25.65	15	19.0	2.62	1.1	6.4	0.024
- Moderate	11	14.1	8	10.1	2.70	0.9	8.7	0.067
- Serious	20	25.65	3	3.8	13.1	3.4	72.9	<0.0001
Urgent miction	80	31.3	78	14.1	2.78	1.2	6.8	0.01
Trouble in defecation ^†^	80	43.8	78	14.1	4.8	2.1	11.4	<0.0001

^†^ These were constipation, faecal incontinence, faecal emergencies, and no control of the anal sphincter.

Four items were validated with the Cronbach alpha coefficient (*alpha* = 0,847; 95% CI = 0,793 – 0,886): blue mood, decreased energy, decreased of pep and severe asthenia. The exposed subjects had a score of 2.6, significantly greater than 0.8 in the non exposed subjects (t test = 8.24, df = 147.7, p < 0.0001).

### Sequelae associated with meningitis

Among the 132 subjects remaining at the end of follow-up, there was no gender predominance with a sex ratio M:F = 0.94. The mean age was 12.8 years (SD = 8.1 years, range = 5 – 55 years), 13 and 12.6 years respectively for exposed and non exposed subjects.

Three functional symptoms persisted: headaches, asthenia, and vertigo among respectively 31.3, 36.9, and 22.4% of the exposed subjects with a significant risk of persistence.

The risk of motor impairment reached 7.8 among the subjects exposed to meningitis compared to the non exposed subjects. A motor impairment was significantly retrieved among 12.3% of the exposed versus 1.6% of the non exposed subjects. Hearing loss disabled 31.3% of the exposed subjects and 10.4% exhibited a serious deafness with a significant risk of 7.1. When combining motor impairment and hearing loss, the risk of neurological sequelae was 1.8 for exposed subjects versus non exposed subjects (Table 3).

**Table 3:** Frequency and risk of sequelae after being exposed to an acute bacterial meningitis episode

**Sequelae**	**Exposed**		**Non exposed**		**p**	**Crude RR**	**95% CI RR**	
	**n = 67**	**%**	**n = 65**	**%**				
Functional symptoms								
Headache	67	31.3	65	13.8	0.022	2.3	1.1	4.6
Asthenia	65	36.9	63	12.7	0.002	2.9	1.4	6.0
Vertigo	67	22.4	65	7.7	0.027	2.9	1.1	7.6
Hearing loss assessment^1^								
Bone conduction altered or mixed	64	7.8	65	7.7	1	1.2	0.4	3.8
All levels of hearing loss	67		65					
- No	46	68.7	53	81.5				
- Mild	8	11.9	8	12.3	0.8	1.1	0.5	2.8
- Moderate	6	9.0	3	4.6	0.31	2.2	0.6	8.2
- Serious	7	10.4	1	1.5	0.031	7.1	0.9	56.0
- Moderate/serious	13	19.4	4	6.2	0.034	3.1	1.1	9.1
Motor impairment^2^	65	12.3	64	1.6	0.033	7.8	1	60.2
Neurological^3^	66	36.4	64	20.3	0.053	1.79	1.0	3.2

^1^ The most affected ear was retained.

^2^ Concerning walking and tonus.

^3^ Hearing loss or motor impairment.

No behavioural troubles persisted as the score was similar among the exposed and non exposed subjects, 0.9 versus 0.5 (t test = 1.75, df = 125.6, p = 0.08).

## Discussion

Three types of sequelae were observed in this study: functional symptoms, hearing loss, and motor impairment. We did not however address mental retardation due to the lack of standard intelligence scoring tests adapted to a country with low literacy rates. Functional symptoms like headaches and asthenia were observed at frequencies of 31.3 and 36.9%. An alteration of the VIII^th^ pair of cranial nerve with vertigo, at least moderate and serious hearing loss was developed respectively by 22.4, 19.4 and 10.4% of the exposed subjects. Finally, motor impairment was observed among 12.3% of exposed subjects. The frequencies of these sequelae are among the highest values retrieved in studies performed in Africa [4]. Generally, the frequency of sequelae increases with the severity of the clinical form that is usually found among pneumococcus cases. As *N*. *meningitidis* W was the major causal agent in our study, it raises a question on the severity linked to this serogroup which had a case fatality ratio of 12% estimated in a study performed between 2003 and 2006 in Niger [11]. Another possible explanation for the high frequencies of sequelae observed in our study is the short duration of the follow-up, three months in average, which could lead to an over estimation because some sequelae could spontaneously regress after this delay. However, increasing the study duration could lead to a higher proportion of subjects lost to follow-up and introduce a strong bias in the estimation of frequencies and risks. Nevertheless, the Société de Pathologie Infectieuse de Langue Française recommends to perform an audiogram and a neurological examination one month after acute meningitis infection, followed by a clinical surveillance of the audition every three months during one year [23].

The frequency of severe/profound hearing loss was 10.4% compared to 1.6% in a study performed in Ghana [2]. A high frequency of severe/profound hearing loss was found in a study performed in Malawi among hospitalised subjects [24]. Variations in frequencies observed in the different studies could be related to the severity of the meningitis since a severe illness could exhibit a higher risk of sequelae [25].

Persistent headaches were declared more than one week after CSF was taken and were therefore not linked to the lumbar puncture [26,27]. This symptom was usually found at a similar frequency in the national surveillance system of Sweden [28]. At the end of follow-up, exposed subjects had a significantly higher spontaneous declaration of headaches (27.9%) and psychological troubles (16.2%) than non exposed subjects (21.5% and 3.1% respectively, data not shown). This result should incite a better assessment of functional symptoms like headaches by investigating the type, intensity, irradiation, enhancing or calming factors, and familial predisposition. Although they were declared significantly more by the exposed subjects, psychological troubles were not considered as sequelae after assessment by the psychologists even though they were frequent at the inclusion, as shown using a reliable and validated score (Cronbach alpha coefficient > 0.8). These results are in accordance with Sumpter *et al*. (2011) who concluded that psychological consequences are due to sequelae after meningitis and not directly due to meningitis [29].

An important asthenia persisted among 36.9% of the exposed subjects, a frequency close to the 40.1% observed in a study performed in Ghana [2]. This symptom seems to be of important interest as it is frequently found and was retained in the score, though not more frequently declared spontaneously by the exposed subjects compared to the non exposed subjects (data not shown). These discordant observations should orientate researchers’ attention to the assessment of subjective psychological troubles to be probably explored more adequately using qualitative study designs [30].

One of the limits of this study could be its power which restricted the identification of other sequelae such as epilepsy and cecity or visual loss observed in other studies [4,26]. Epilepsy could occur after several years of evolution whereas our study had only three months of follow-up [31]. This duration of follow-up was chosen according to the protocol study validated by the scientific committee of the study and due to difficulties encountered on the field such as health care accessibility which could inflate the percentage of participants lost to follow-up. In addition, it was difficult to collect clinical data relative to treatment because health care centres in Niger were insufficiently or not inconsistently supplied with antiepileptic drugs or corticoids; these drugs could play a role in the occurrence of sequelae after meningitis. Acute neurological signs like hydrocephalus were investigated, but none was observed. Another limit was the number of subjects lost during follow-up that could introduce a non differential bias of classification and overestimate the relative risks. A subject loss to follow-up of more than 15% was observed and could be explained by subjects living in remote areas with no means to come to appointments for follow-up. After taking into account lost subjects by supposing they were finally found disabled at the control visit, meningitis remained a significant risk factor only for asthenia (data not shown).

The high mortality rates due to bacterial meningitis in Niger have enhanced the surveillance and control strategies based on the implementation of reactive vaccination campaigns and early antibiotherapy. Recently, the introduction of a conjugate vaccine increased the hope of eradicating meningococci of serogroup A, one of the major causes of large epidemics in the meningitis belt. Nevertheless, pneumococci and meningococci of other serogroups (*i*.*e*.: X and W) still threaten the populations of the meningitis belt. This study carried out in Niger confirms two serious neurological sequelae occurring after meningitis: high frequencies of severe and profound hearing loss, and motor impairment. Ideal follow-up of patients requires the most modern radiographic imageries, not affordable by Niger, for the timely detection of labyrinthitis ossificans [32]. Cochlear implantation is indicated in the first two weeks of meningitis in case of deafness [33]. Again, this treatment is not affordable to Niger’s populations. Similarly, hearing aids cost 20 times higher in developing countries than when purchased elsewhere [34].

## Conclusions

Neurological sequelae, particularly severe hearing loss and motor impairment, occurring after bacterial meningitis in Niger should sensitize African public health authorities towards early and rapid patient management accompanied by the development of appropriate rehabilitation measures through existing strategies and guidelines to manage hearing loss [35,36]. Continuously implementing and improving early vaccination campaigns to prevent outbreaks remains a big challenge and a way to prevent sequelae especially with the emergence of W in Niger and other Sahelian countries, since 2010.

## Competing interests

The authors declare that they have no competing interests.

## Authors’ contributions

JFJ and ZT contributed to conception and design of data. JFJ performed the analysis and interpretation of data. AAY made substantial contributions to acquisition of data and interpretation of data. JMC coordinated the microbiological surveillance in Niger. JFJ drafted the manuscript. All the authors read and approved the final manuscript and gave final approval of the version to be published.

## Pre-publication history

The pre-publication history for this paper can be accessed here:

http://www.biomedcentral.com/1471-2334/13/228/prepub

## References

[B1] Tikhomirov E Santamaria M Esteves K Meningococcal disease: public health burden and control World Health Stat Q 1997 50 3–4 170 177 9477545

[B2] Hodgson A Smith T Gagneux S Akumah I Adjuik M Pluschke G Binka F Genton B Survival and sequelae of meningococcal meningitis in Ghana Int J Epidemiol 2001 30 6 1440 1446 10.1093/ije/30.6.1440 11821360

[B3] Oostenbrink R Maas M Moons KG Moll HA Sequelae after bacterial meningitis in childhood Scand J Infect Dis 2002 34 5 379 382 10.1080/00365540110080179 12069024

[B4] Ramakrishnan M Ulland AJ Steinhardt LC Moisi JC Were F Levine OS Sequelae due to bacterial meningitis among African children: a systematic literature review BMC Med 2009 7 47 10.1186/1741-7015-7-47 19751516 PMC2759956

[B5] Edmond K Clark A Korczak VS Sanderson C Griffiths UK Rudan I Global and regional risk of disabling sequelae from bacterial meningitis: a systematic review and meta-analysis Lancet Infect Dis 2010 10 5 317 328 10.1016/S1473-3099(10)70048-7 20417414

[B6] Goetghebuer T West TE Wermenbol V Cadbury AL Milligan P Lloyd-Evans N Adegbola RA Mulholland EK Greenwood BM Weber MW Outcome of meningitis caused by Streptococcus pneumoniae and Haemophilus influenzae type b in children in The Gambia Trop Med Int Health 2000 5 3 207 213 10.1046/j.1365-3156.2000.00535.x 10747284

[B7] Greenwood B Manson lecture. Meningococcal meningitis in Africa Trans R Soc Trop Med Hyg 1999 93 4 341 353 10.1016/S0035-9203(99)90106-2 10674069

[B8] Meningococcal meningitishttp://www.who.int/mediacentre/factsheets/fs141/en

[B9] Decosas J Koama JB Chronicle of an outbreak foretold: meningococcal meningitis W135 in Burkina Faso Lancet Infect Dis 2002 2 12 763 765 10.1016/S1473-3099(02)00455-3 12467693

[B10] World Health Organisation Meningitis in Chad, Niger and Nigeria: 2009 epidemic season Wkly Epidemiol Rec 2010 85 8 47 63 20210043

[B11] Boisier P Mainassara HB Sidikou F Djibo S Kairo KK Chanteau S Case-fatality ratio of bacterial meningitis in the African meningitis belt: we can do better Vaccine 2007 25 Suppl 1 A24 A29 17521784 10.1016/j.vaccine.2007.04.036

[B12] Collard JM Maman Z Yacouba H Djibo S Nicolas P Jusot JF Rocourt J Maitournam R Increase in Neisseria meningitidis serogroup W135, Niger, 2010 Emerg Infect Dis 2010 16 9 1496 1498 10.3201/eid1609.100510 20735947 PMC3294991

[B13] Merchant SN Gopen Q A human temporal bone study of acute bacterial meningogenic labyrinthitis Am J Otol 1996 17 3 375 385 8817013

[B14] Vienny H Despland PA Lutschg J Deonna T Dutoit-Marco ML Gander C Early diagnosis and evolution of deafness in childhood bacterial meningitis: a study using brainstem auditory evoked potentials Pediatrics 1984 73 5 579 586 6718112

[B15] Douglas SA Sanli H Gibson WP Meningitis resulting in hearing loss and labyrinthitis ossificans - does the causative organism matter? Cochlear Implants Int 2008 9 2 90 96 10.1002/cii.344 18246540

[B16] Holborow C Martinson F Anger N A study of deafness in West Africa Int J Pediatr Otorhinolaryngol 1982 4 2 107 132 10.1016/0165-5876(82)90087-8 7129783

[B17] Obiako MN Profound childhood deafness in Nigeria: a three year survey Ear Hear 1987 8 2 74 77 10.1097/00003446-198704000-00003 3582806

[B18] Brobby GW Causes of congenital and acquired total sensorineural hearing loss in Ghanaian children Trop Doct 1988 18 1 30 32 3341087 10.1177/004947558801800112

[B19] Bacterial meningitis (including Haemophilus influenzae type b (Hib), Neisseria meningitidis, and Streptococcus pneumoniae) http://www.who.int/immunization_monitoring/diseases/meningitis_surveillance/en/index.html

[B20] Wait JW Stanton L Schoeman JF Tuberculosis meningitis and attention deficit hyperactivity disorder in children J Trop Pediatr 2002 48 5 294 299 10.1093/tropej/48.5.294 12405172

[B21] Abramson JH WINPEPI updated: computer programs for epidemiologists, and their teaching potential Epidemiol Perspect Innov 2011 8 1 1 10.1186/1742-5573-8-1 21288353 PMC3041648

[B22] Moret L Mesbah M Chwalow J Lellouch J Internal validation of a measurement scale: relation between principal component analysis, Cronbach’s alpha coefficient and intra-class correlation coefficient Rev Epidemiol Sante Publique 1993 41 2 179 186 8493397

[B23] Société de Pathologie Infectieuse de Langue Française; Collège des Universitaires des Maladies Infectieuses et Tropicales (CMIT); Association Pédagogique Nationale pour l’Enseignement de la Thérapeutique (APNET); Société Française de Microbiologie (SFM); Société Française de Médecine d’Urgence (SFMU); Société Française de Neurologie (SFN); Société Française d’ORL (SFORL); Société Française de Pédiatrie (SFP); Société Nationale Française de Médecine Interne (SNFMI); Société de Réanimation de Langue Française (SRLF) **Management of acute community-acquired bacterial meningitides, except in newborn infants. Short text. November 2008. Société de pathologie infectieuse de langue française** Rev Neurol (Paris) 2009 165 F205 F216 20222183

[B24] Forsyth H Kalumbi F Mphaka E Tembo M Mwenechanya J Kayira K Bwanaisa L Njobvu A Walsh A Molyneux E Hearing loss in Malawian children after bacterial meningitis: incidence and risk factors Audiological Medicine 2004 2 2 100 107 10.1080/16513860410033711

[B25] Davey PG Jabeen FJ Harpur ES Shenoi PM Geddes AM A controlled study of the reliability of pure tone audiometry for the detection of gentamicin auditory toxicity J Laryngol Otol 1983 97 1 27 36 10.1017/S0022215100093762 6337228

[B26] Kuntz KM Kokmen E Stevens JC Miller P Offord KP Ho MM Post-lumbar puncture headaches: experience in 501 consecutive procedures Neurology 1992 42 10 1884 1887 10.1212/WNL.42.10.1884 1407567

[B27] Lowery S Oliver A Incidence of postdural puncture headache and backache following diagnostic/therapeutic lumbar puncture using a 22G cutting spinal needle, and after introduction of a 25G pencil point spinal needle Paediatr Anaesth 2008 18 3 230 234 10.1111/j.1460-9592.2008.02414.x 18230066

[B28] Berg S Trollfors B Hugosson S Fernell E Svensson E Long-term follow-up of children with bacterial meningitis with emphasis on behavioural characteristics Eur J Pediatr 2002 161 6 330 336 10.1007/s00431-002-0957-1 12029452

[B29] Sumpter R Brunklaus A McWilliam R Dorris L Health-related quality-of-life and behavioural outcome in survivors of childhood meningitis Brain Inj 2011 25 13–14 1288 1295 21961570 10.3109/02699052.2011.613090

[B30] Greenhalgh T Taylor R Papers that go beyond numbers (qualitative research) BMJ 1997 315 740 743 10.1136/bmj.315.7110.740 9314762 PMC2127518

[B31] Salih MA Khaleefa OH Bushara M Taha ZB Musa ZA Kamil I Hofvander Y Olcen P Long term sequelae of childhood acute bacterial meningitis in a developing country. A study from the Sudan Scand J Infect Dis 1991 23 2 175 182 10.3109/00365549109023397 1853165

[B32] Isaacson B Booth T Kutz JW Jr Lee KH Roland PS Labyrinthitis ossificans: how accurate is MRI in predicting cochlear obstruction? Otolaryngol Head Neck Surg 2009 140 5 692 696 10.1016/j.otohns.2008.12.029 19393413

[B33] Tinling SP Colton J Brodie HA Location and timing of initial osteoid deposition in postmeningitic labyrinthitis ossificans determined by multiple fluorescent labels Laryngoscope 2004 114 4 675 680 10.1097/00005537-200404000-00015 15064623

[B34] Kumar S WHO tackles hearing disabilities in developing world Lancet 2001 358 9277 219 11476854 10.1016/S0140-6736(01)05460-5

[B35] Lasisi OA Ayodele JK Ijaduola GT Challenges in management of childhood sensorineural hearing loss in sub-Saharan Africa, Nigeria Int J Pediatr Otorhinolaryngol 2006 70 4 625 629 10.1016/j.ijporl.2005.08.009 16168496

[B36] Tucci D Merson MH Wilson BS A summary of the literature on global hearing impairment: current status and priorities for action Otol Neurotol 2010 31 1 31 41 10.1097/MAO.0b013e3181c0eaec 20050266

